# Confidence interval approach for evaluating bias in laboratory methods

**DOI:** 10.1155/S1463924688000276

**Published:** 1988

**Authors:** K. F. Yee

**Affiliations:** Beecham Pharmaceuticals Coldharbour Road The Pinnacles Essex Harlow CM19 5AD UK

## Abstract

A statistically significant difference in mean values between two laboratory quantitation methods is interpreted as a bias. Sometimes such a difference is so minute that it does not constitute any practical concern. An alternative approach is to test statistically whether the two methods are close enough, not for equality. This is to look at the confidence interval of the mean method difference and does not entail any additional statistical tests.


Journal of Automatic Chemistry, Vol. 10, No. 3 (July–September 1988), pp. 144–146

### Confidence interval approach for evaluating bias in laboratory methods

**K. F. Yee**

*Beecham Pharmaceuticals, Coldharbour Road, The Pinnacles, Harlow, Essex CM19 5AD, UK*

*A statistically significant difference in mean values between two laboratory quantitation methods is interpreted as a bias. Sometimes such a difference is so minute that it does not constitute any practical concern. An alternative approach is to test statistically whether the two methods are close enough, not for equality. This is to look at the confidence interval of the mean method difference and does not entail any additional statistical tests.*

### Introduction

When comparing a new laboratory quantitation method to a standard method, the new method ideally should give the same, if not more accurate, results than the standard method. Any systematic difference between the two methods is called a bias. The conventional statistical evaluation of bias is by testing the null hypothesis that the quantitation result from the new method equals that of the standard one. The methodology for comparing two means is given in most statistical references, for example see Snedecor and Cochran, chapter 4 [1].

One concludes that the new method is biased if the test statistic is significant at the $\alpha$-level, where $\alpha$ is the type I error chosen by the investigator, for example $\alpha = 0{\cdot}05$. The test statistic is usually of the form:

$$\mathrm{d}/\mathrm{SE}(\mathrm{d}) \qquad (1)$$

where $d$ is the mean method difference and $\mathrm{SE}(d)$ is the standard error of $d$.

Sometimes a statistically significant difference, $d$, is so small in magnitude that it does not materially affect the quantitation of samples. Such a difference is not meaningful in laboratory practice.

Indeed, one can argue that because the new and standard methods are not identical, given enough samples, one can always demonstrate that there is a bias with the new method. The new method is penalized because of the high precision in our statistical evaluation process. An alternative approach in statistical evaluation is clearly needed in this instance.

### Method

The idea of testing two mean values for being similar but not necessarily identical is not new. In the pharmaceutical industry, two formulations of a drug are said to be bioequivalent if their mean values with respect to some clinical or pharmacokinetic parameter are close enough [2]. The same concept can be applied to comparing two laboratory quantitation methods: two methods are said to be 'equivalent' if their mean difference is less than a prescribed quantity, say $H$, which is called the maximum acceptable difference. $H$ could be chosen from experience or be a value deemed practical by the investigator.

Statistically one accepts the equivalence of two laboratory methods at the $\alpha$-level if the $(1\text{-}\alpha)$ 100% confidence interval of the mean method difference d, say $(C_1, C_2)$, is completely contained in the interval $(^{-}H, H)$, i.e.

$$-H < C_1 < d < C_2 < H \qquad (2)$$

If the method difference is assumed to be normally distributed then the test statistic (equation [1]) is a $t$-distribution, and the $(1\text{-}\alpha)100\%$ confidence interval of $d$ is:

$$C_1, C_2 = d \pm t_{\alpha/2,\nu}\, \mathrm{SE}(d) \qquad (3)$$

where $_\nu$ is the degrees of freedom of $\mathrm{SE}(d)$ and $t_{\alpha/2,\,\nu}$ is the $(1\text{-}\alpha/2)$- 100 percentile of the $t$-distribution with $\nu$ degrees of freedom.

The above test procedure is also applicable when there is only one laboratory method. Sometimes one wants to test whether the laboratory method is accurate enough with respect to a known target value, say $T$. Let $X$ be the mean value of the laboratory method, then $d = X - T$ and $\mathrm{SE}(d) = \mathrm{SE}(X)$, and the test procedure is the same as described above. For example the laboratory method is a HPLC assay measuring the recovery of a chemical entity. The natural target value ($T$) will be 100% in this instance.

The two methods case can sometimes be reduced to a one method situation. If the ratio, instead of the difference, of the two methods is being investigated and assumed to be Normally distributed, then $X =$ mean ratio and the target $T = 100\%$ again. This situation will be illustrated in the next section.

### Example

To illustrate the test of equivalence, an example has been taken from Griffiths *et al.* [3]: the potassium levels of 21 patient serum specimens were analysed by Beckman Astra-8 and flame photometry methods. The Astra method was arbitarily defined as the standard method, and the flame photometry as the new method. The ratio of the new method to the standard method (expressed in %) was assumed to be normally distributed and was used to evaluate bias of the new method. The raw data and the ratio are reproduced in table 1.

*Table 1. Raw data for potassium by two quantitative methods and their ratio (see Griffiths et al. [3]).*

| Sample | Astra | Flame | % |
|---|---|---|---|
| 1 | 2·4 | 2·4 | 100·0 |
| 2 | 4·8 | 4·8 | 100·0 |
| 3 | 4·0 | 4·0 | 100·0 |
| 4 | 4·6 | 4·7 | 102·2 |
| 5 | 3·9 | 3·9 | 100·0 |
| 6 | 4·1 | 4·2 | 102·4 |
| 7 | 3·8 | 3·8 | 100·0 |
| 8 | 3·2 | 3·3 | 103·1 |
| 9 | 4·6 | 4·6 | 100·0 |
| 10 | 2·9 | 3·0 | 103·4 |
| 11 | 4·9 | 5·0 | 102·0 |
| 12 | 3·5 | 3·6 | 102·9 |
| 13 | 4·8 | 4·9 | 102·1 |
| 14 | 3·7 | 3·8 | 102·7 |
| 15 | 3·8 | 3·9 | 102·6 |
| 16 | 4·5 | 4·6 | 102·2 |
| 17 | 4·2 | 4·2 | 100·0 |
| 18 | 4·2 | 4·2 | 100·0 |
| 19 | 4·3 | 4·4 | 102·3 |
| 20 | 3·9 | 4·0 | 102·6 |
| 21 | 3·3 | 3·4 | 103·0 |
| Mean | | | 101·6 |
| SD | | | 1·33 |
| SE of mean | | | 0·29 |
| 95% confidence | | | 101·0 |
| Interval of mean | | | 102·2 |

The target value $T = 100\%$. From table 1, $d = 101{\cdot}6–100 = 1{\cdot}6\%$, $SE(d) = 0{\cdot}29\%$. In the conventional significance test approach, the test statistic:

$$d/SE(d) = 1{\cdot}6/0{\cdot}29 = 5{\cdot}52 \text{ with 20 degrees of freedom}$$

was highly significant (the critical value at $\alpha = 0{\cdot}05$ and 20 degrees of freedom is $t_{0{\cdot}025,20} = 2{\cdot}086$), i.e. the flame photometry was biased in that its measurement was on average 1·6% higher than that of the Astra method.

However, if one feels that only method differences exceeding a certain level constitute meaningful difference, then the test of equivalence approach will be more appropriate. From equation (3) the 95% confidence interval of $d$: $1{\cdot}6 \pm 2{\cdot}086 \times 0{\cdot}29 = (1{\cdot}0, 2{\cdot}2)$. Various levels of the maximum acceptable difference, $H$, have been chosen to demonstrate the interpretation of the test of equivalence at $\alpha = 0{\cdot}05$. Figure 1 illustrates these situations.

(1) $H = 0{\cdot}5$
The 95% confidence interval of $d$ was completely outside the interval $(-0{\cdot}5, 0{\cdot}5)$. The flame photometry method gave significantly higher results than the Astra method by 0·5% or more. The conclusion is the same as the conventional significance test in this instance, i.e. not equivalent.

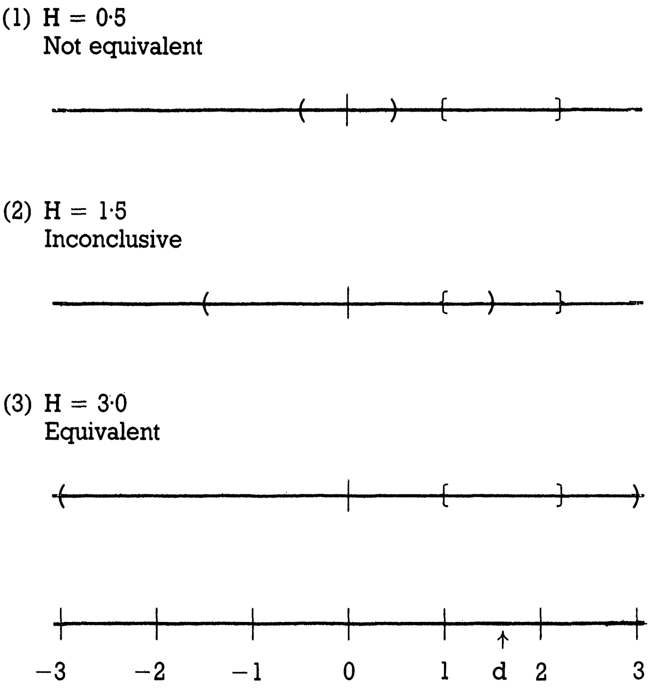

*Figure 1. The test of equivalence between the Astra and Flame methods for various maximum acceptable differences, H. { } is the 95% confidence interval of the mean difference d.*

(2) $H = 1{\cdot}5$
The 95% confidence interval of $d$ overlapped the interval $(-1{\cdot}5, 1{\cdot}5)$. There was inconclusive evidence to discern the equivalence of the two methods, one way or the other. It indicates that more samples are required to reach any conclusion at this significance level.

(3) $H = 3{\cdot}0$
The 95% confidence interval of $d$ was completely contained in the interval $(-3,3)$. The flame photometry method was equivalent to the Astra method in that the results of each differed from the other by less than 3%.

### Discussion

One can argue that there is always a bias between two laboratory quantitation methods. A statistically significant difference between methods is of acadmic interest only, unless the magnitude of the difference also has a practical implication. A dogmatic application of the conventional significance test might not give a meaningful interpretation. In contrast, the test of equivalence provides wider scope, and sometimes a more realistic approach in comparing two methods. No additional statistical test is needed in this evaluation procedure.

The choice of the maximum acceptable difference, $H$, is crucial in the successful application of the test of equivalence. This is not a statistical decision, but one which must be determined from experience, or a value which is deemed meaningful from the practitioner's perspective.

When there is inconclusive evidence to detect whether two methods are equivalent, more samples are needed in the test. The methodology for determining the optimal sample size in a comparative experiment using the conventional significance test is well known [1], but the calculation is far more complex in the equivalence test context [4], and is beyond the scope of this note.

So far only the case where data are normally distributed has been illustrated. There is no difficulty in applying the equivalence test idea to data having other types of distribution so long as the confidence interval of the mean method difference can be obtained.

### Acknowledgement

The work was done while I was at Syntex Research Centre, Edinburgh, UK.

### References

1. Snedecor, G. W. and Cochran, W. G., *Statistical Methods* (The Iowa State University Press, Iowa, 1976), p. 91.
2. Metzler, C. M., *Biometrics,* **30** (1974), 309.
3. Griffiths, W. C., Camara, P., Diamond, I. and Pezzullo, J. C., *Journal of Automatic Chemistry,* **8** (1986), 147.
4. Yee, K. F., *Biometrics,* **42** (1986), 961.

### INDUSTRY SCOREBOARD—1988

A world-wide ranking of analytical instrument firms has recently been published by the *Analytical Instrument Industry Report.* Full copies of the analysis, which broadly estimates sales of product and service to analytical laboratories in 1987 for 165 companies, are available from *Gordon Wilkinson, AIIR, PO Box 78, East Grinstead RH19 2YW, UK.* The following is an extract from *AIIR*'s analysis:

Where leading companies disclose analytical product group sales, this usually includes instruments, accessories, supplies, data systems, and service revenues.

*AIIR* therefore included a selection of smaller companies that also make these supplies. Your familiarity with the industry will enable you to assess the relevant business areas in which these firms compete. *AIIR*'s definition of analytical instrumentation includes surface analysis and electron-optic products.

Note: companies are arranged in alphabetical order in each range.

| | |
|---|---|
| **400–450** | Perkin-Elmer (USA) |
| **350–400** | Hewlett-Packard (USA) |
| | Shimadzu (Japan) |
| **250–300** | JEOL (Japan) |
| | Philips Analytical (The Netherlands) |
| **200–250** | Hitachi (Japan) |
| | Pharmacia/LKB (Sweden) |
| | Waters Chromatography Division/Millipore (USA) |
| **150–200** | Rigaku (Japan) |
| | Varian (USA) |
| **100–150** | Bruker (FR Germany) |
| | Finnigan (USA) |
| | Fisons, including ARL, CEST, J&W (UK) |
| | VG Instruments [SEM + MS] (UK) |

These industry leaders currently account for almost $3 billion worth of sales. Of this group, five are US-owned, five are European and four are Japanese.

Japanese firms again have relatively higher rankings this year, primarily because of the continuing appreciation of the yen against the dollar since September 1985. In real terms, however, their sales have been badly hit in the past year.

Companies with sales in the $40 million to $100 million range appear in the following table. Of these, 16 are US-owned, six are European, and four are Japanese.

*Report* continued with $40–100 M rankings on page 157

